# Multicentre cluster randomised controlled trial evaluating implementation of a fire prevention Injury Prevention Briefing in children’s centres: study protocol

**DOI:** 10.1186/1471-2458-14-69

**Published:** 2014-01-22

**Authors:** Toity Deave, Elizabeth Towner, Elaine McColl, Richard Reading, Alex Sutton, Carol Coupland, Nicola Cooper, Jane Stewart, Mike Hayes, Emma Pitchforth, Michael Watson, Denise Kendrick

**Affiliations:** 1 Centre for Child & Adolescent Health, Health & Life Sciences, University of the West of England Bristol, Oakfield House, Oakfield Grove, Bristol BS8 2BN, UK; 2 Newcastle Clinical Trials Unit, 4th Floor, William Leech Building, The Medical School, Newcastle University, Framlington Place, Newcastle upon Tyne NE2 4HH, UK; 3 Jenny Lind Paediatric Department, Norfolk and Norwich University Hospital, Colney Lane, Norwich NR4 7UY, UK; 4 Department of Health Sciences, University of Leicester, Leicester LE1 7RH, UK; 5 Division of Primary Care, School of Medicine, Tower Building, University Park, Nottingham NG7 2RD, UK; 6 Child Accident Prevention Trust, Canterbury Court (1.09), 1 - 3 Brixton Road, London SW9 6DE, UK; 7 London School of Economics Health, London School of Economics and Political Science, Houghton Street, London WC2A 2AE, UK; 8 School of Health Sciences, D86, Queen’s Medical Centre, University of Nottingham, Nottingham NG7 2HA, UK

## Abstract

**Background:**

The UK has one of the highest fatality rates for deaths from fire-related injuries in children aged 0–14 years; these injuries have the steepest social gradient of all injuries in the UK. Children’s centres provide children under five years old and their families with a range of services and information, including home safety, but their effectiveness in promoting injury prevention has yet to be evaluated. We developed a fire prevention intervention for use in children’s centres comprising an Injury Prevention Briefing (IPB) which provides evidence on what works and best practice from those running injury prevention programmes, and a facilitation package to support implementation of the IPB. This protocol describes the design and methods of a trial evaluating the effectiveness and cost-effectiveness of the IPB and facilitation package in promoting fire prevention.

**Methods/Design:**

Pragmatic, multicentre cluster randomised controlled trial, with a nested qualitative study, in four study centres in England. Children’s centres in the most disadvantaged areas will be eligible to participate and will be randomised to one of three treatment arms comprising: IPB with facilitation package; IPB with no facilitation package; usual care (control). The primary outcome measure will be the proportion of families who have a fire escape plan at follow-up. Eleven children’s centres per arm are required to detect an absolute difference in the percentage of families with a fire escape plan of 20% in either of the two intervention arms compared with the control arm, with 80% power and a 5% significance level (2-sided), an intraclass correlation coefficient of 0.05 and assuming outcomes are assessed on 20 families per children’s centre. Secondary outcomes include the assessment of the cost-effectiveness of the intervention, other fire safety behaviours and factors associated with degree of implementation of the IPB.

**Discussion:**

This will be the first trial to develop and evaluate a fire prevention intervention for use in children’s centres in the UK. Its findings will be generalisable to children’s centres in the most disadvantaged areas of the UK and may also be generalisable to similar interventions to prevent other types of injury.

**Trial registration:**

http://NCT01452191 (date of registration: 13/10/2011).

## Background

Globally, injuries are an important cause of morbidity and mortality in childhood [1]. The UK does not compare well with other high-income countries, having one of the highest rates for deaths from fire and flames in children aged 0–14 years [2]. In 2011–12, the English Fire and Rescue Service attended 44,300 house fires with 21 fatalities in those under 16 years with 35 times that number injured [3].

Within countries there are steep social gradients in childhood fire-related deaths and injuries, which disproportionately affect the disadvantaged [4]. In England and Wales, children whose parents had never worked or were long-term unemployed have death rates from exposure to smoke, fire and flames 38 times higher than those of children whose parents had managerial/professional occupations [5].

There is evidence that some interventions are effective in reducing the risk of fire-related injuries and in promoting fire prevention practices. Smoke alarms reduce risk of death in house fires [6,7]. Education, with or without safety equipment being provided, is effective in increasing the prevalence of functioning smoke alarms [8,9] and home safety education increases the prevalence of fire escape planning [8]. Despite this, there is little evidence of the systematic implementation of such injury prevention in the NHS [10] and it is unlikely that this is any different in the social care or the voluntary sectors. A recent systematic review identified the main barriers and facilitators to implementing injury prevention interventions; these included the type of approach used (one-one; group work; partnership working; tailored methods), characteristics of the deliverer, the complexity of the intervention, accessibility to safety equipment and the importance of achieving behavioural change [11]. It is therefore important that interventions to promote injury prevention take these barriers and facilitators into account.

Children’s centres provide community-based integrated services, information and support for families with pre-school children. They aim to improve outcomes for young children and their families, with a particular focus on the most disadvantaged, in order to reduce inequalities in health [12,13]. Home safety is within the remit of children’s centres consequently they have the potential to promote fire prevention and to reach a population at particular risk of fire-related injury. We have therefore developed a fire prevention intervention for use in children’s centres, comprising an Injury Prevention Briefing (IPB) which provides evidence on what works and best practice from those running injury prevention programmes and a facilitation package to support implementation of the IPB. The five key messages in the intervention are: the importance of smoke alarm use and maintenance, having a family fire escape plan, identifying potential causes of house fires, safe storage of matches and lighters and having a bedtime fire safety routine. This protocol describes a trial evaluating the effectiveness and cost-effectiveness of the IPB and facilitation package in promoting fire prevention.

### Objective

The objective of the trial is to evaluate the effectiveness and cost effectiveness of an educationally based intervention (IPB) with or without facilitation, as a means of changing behaviours to improve fire safety in the home.

## Methods/Design

A three-arm multicentre cluster randomised controlled trial, with an economic analysis and a nested qualitative study, set in 4 trial sites in England (Nottingham, Bristol, Norwich and Newcastle). We chose a cluster randomised controlled trial because the intervention will be delivered at the level of children’s centres and to prevent contamination between treatment arms that could have arisen through individual treatment arm allocation of families living in close proximity.

### Participants

#### Children’s centres

Children’s centres were established in phases, with the first phase being those in the most disadvantaged areas. Children’s centres in the four trial sites will be eligible to participate if their catchment area has more than 50% of under-5 year-old children living in one of the 30% most disadvantaged Super Output Areas in England [14]. Children’s centres will be invited to take part by post followed by a telephone call and a face-to-face visit at which a consent form will be completed by the children’s centre manager and baseline data will be obtained. Priority will be given to phase one children’s centres who will be invited to participate first, and invitations will be extended to phase two children’s centres if the sample size requirements are not fulfilled with phase one centres.

### Families

Families who have attended the participating children’s centre in the previous three months, who have a child under three years old and who live within the catchment area of that children’s centre will be eligible to participate. Families where a parent is under 16 years of age will be excluded from participation.

Families will be recruited using a range of strategies including:

1. Trial information is sent by post;

2. Children’s centre staff approach families face-to-face either in the centre or in the family home and provide information;

3. Children’s centre staff introduce families to a member of the research team who explains the trial and provides trial information.

Those agreeing to take part will complete a consent form and baseline questionnaire, either at the children’s centre or at home and return them by post. Participants will only be considered to be recruited to the trial if they complete a consent form and the baseline questionnaire.

### Intervention

The intervention will comprise an IPB, bringing together information from the scientific literature on effectiveness and best practice in fire prevention from people who run injury prevention programmes in the field and a package to facilitate its implementation. The intervention was developed using the UK Medical Research Council (MRC) guidance for the development and evaluation of complex interventions [15] and included the following stages:

1. Identifying the evidence base.

Evidence about the effectiveness of interventions was ascertained from systematic reviews of interventions to prevent injuries from house fires [8,9,16]; facilitators and barriers for home injury prevention interventions for pre-school children [11] and decision-analyses related to the prevention of house fires [17].

Evidence about the design, content and delivery of the intervention came from several sources. These included the Health Development Agency [18] 'Effective Action Briefing’ for putting evidence into practice for the promotion of domestic smoke alarms [19] and a review of the literature on the implementation and facilitation of health promotion interventions undertaken as preliminary work for this trial. This identified one framework which we used to guide the design and evaluation of the facilitation package: the PARIHS framework (Promoting Action on Research in Health Services) [20] and Carroll’s fidelity framework [21] which we used to measure the fidelity of the intervention. The PARIHS framework provides three interacting core elements of evidence, context and facilitation and the Carroll framework informs measurement of fidelity in terms of adherence to an intervention; exposure or dose; quality of delivery; participant responsiveness and programme differentiation.

2. Identifying appropriate theory.

We developed our intervention based on factors thought to explain most health-related behaviours across five behavioural change theories comprising the health belief model, social cognitive theory, the theory of reasoned action, the theory of self-regulation and self-control and the theory of subjective culture and interpersonal relations, as described in the review of behaviour change theories for injury prevention by Gielen and Sleet [22]. Three factors were described as necessary and sufficient for producing a behaviour:

a. The person forms a strong positive intention or makes a commitment to perform the behaviour;

b. There are no environmental barriers that make it impossible to perform the behaviour;

c. The person possesses the skills necessary to perform the behaviour.

Our intervention therefore aimed to help individual participants (both children’s centre staff and families) form intentions to change behaviour, remove environmental barriers and to provide participants with the skills to perform the behaviour.

3. Modelling processes and outcomes.

We interviewed national and local leaders who had responsibility for policy and operational matters related to children’s centres. We undertook four workshops, one in each trial site, with local practitioners and policy makers, including children’s centre managers and staff, the Fire and Rescue Service, NHS staff and commissioners of children’s services. These were designed to allow input from potential end-users and those with specialist expertise in implementation to the design of the IPB and facilitation package. They were also used to ensure the IPB complemented and recognised existing fire prevention initiatives, built on existing knowledge about how to implement programmes in children’s centres and how to reach families in the community. We used postal surveys [23], interviews with children’s centre managers and staff and structured interviews with parents attending children’s centres [24] to develop an understanding of current injury prevention activities, including parents’ understanding and use of fire escape plans. We also used interviews with parents of pre-school children to explore barriers and facilitators to injury prevention.

### Allocation to intervention and delivery of intervention

Once the required number of families have been recruited at a children’s centre, the children’s centre will be randomised to one of three arms: IPB plus facilitation, IPB only or usual care (control arm) (see below for method of randomisation). The intervention will be delivered over 12 months.

Children’s centres in the IPB plus facilitation arm will be given the following:

a) The fire prevention IPB containing (i), information for children’s centre managers on why preventing fire-related injuries is important, who the target group is, what interventions can be provided, creative ways of reaching the target group and how to evaluate their use of the IPB and (ii), information for children’s centre staff on why preventing fire-related injuries is important, who is at greatest risk, what are the main causes of house fires, what staff can do to help, what works to prevent house fires, where to get specialist advice and help and examples of activities they can do with parents, including session plans and resources covering smoke alarms, fire escape plans, the causes of house fires, children’s development and behaviour and risk of house fires and bedtime safety routines. Children’s centres will be asked to deliver the fire prevention messages to families participating in the trial over a twelve month period, using whatever format they think is most appropriate for their families. If they are unable to deliver all messages, they are asked to focus on smoke alarms and fire escape plans as these are the most evidence based.

b) A training session will be provided by the Child Accident Prevention Trust and the research team focussed on the IPB and its potential use within a children’s centre setting. This comprises an introduction to children’s injuries in general and fire-related injuries in particular; the development, principles and content of the IPB, opportunities to practice using the IPB and to develop an outline plan for how the IPB might be implemented and a discussion of data collection requirements for the trial.

*Facilitation contacts* at month 1, 3 and 8 will use a two-stage approach comprising a postal or electronic questionnaire followed by a face-to-face or telephone interview, depending on progress with implementing the IPB. A fourth contact will be made at months 4 to 5 if there is no progress with implementation of the IPB at month 3. The facilitation contacts will collect information on the progress of IPB implementation, staff engagement, consistency of delivery, address questions and discuss barriers to implementation, give advice and examples of good practice from other centres and provide a resource list, contact with other organisations and address other questions or concerns. To maximise potential usability of the intervention after the trial, the facilitation package was designed to be similar to the advice and support that might be provided by an injury prevention coordinator (as recommended in NICE guidance on injury prevention [25]).

IPB only arm: the IPB will be posted to children’s centres and no training or facilitation will be provided.

Control arm: children’s centres will continue to provide their usual information on home safety. The IPB will be posted to children’s centres after collection of post-intervention data.

### Outcome measures

#### Definition of primary and secondary outcome measures

The primary outcome will be the proportion of families who report that they have a fire escape plan at 12 months follow-up.

Secondary outcome measures will include:

### Family participants

1. The proportion of families with a high level of fire escape planning behaviour (using a binary measure of fire escape plan behaviour derived from five component items using latent variable analysis. The component items are: having door keys accessible, having window lock keys accessible, having a torch beside the bed, knowing the sound of a smoke alarm and having exits clear.);

2. The proportion of families with smoke alarms fitted and working on every level of their home;

3. The proportion of families who report fire setting or match play by their children;

4. A bedtime fire safety routine score;

5. The proportion of families who have accessed smoking cessation services;

6. Number of correct responses to fire safety knowledge questions;

7. The proportion of families who were fairly satisfied or very satisfied with home safety information provided by children’s centres;

8. Implementation of the IPB assessed by:

a. The proportion of families who received advice on each of the 5 key messages in the IPB in the last year;

b. The proportion of families who attended a fire safety session in the last year;

c. The number of fire safety sessions attended by families in the last year;

d. The proportion of families who attended a fire safety session at a children’s centre in the last year;

e. The proportion of families who attended sessions about each of the 5 key messages in the IPB in the last year;

9. Families’ resource-use and expenditure in relation to fire safety practices.

### Children’s centres as participants

10. The proportion of children’s centres providing information and advice on fire prevention;

11. Resource-use and expenditure incurred in relation to fire safety practices in all three arms;

12. Reported implementation of the IPB within children’s centres;

13. The identification of barriers and facilitators to children’s centres implementing the IPB.

All outcome measures will be ascertained at 12 months follow-up, defined as 12 months post commencement of the intervention in the IPB plus facilitation and IPB only arms and 12 months post randomisation in the control arm.

### Ascertainment of outcomes

Outcomes measured at the level of families and children’s centres will be ascertained using a range of tools delivered as summarised in Table 1 and described below.

**Table 1:** Tools for measuring parent and children’s centre outcomes by treatment arm

**Data collection tool**	**IPB plus facilitation arm**	**IPB only arm**	**Control arm**
*Data collected from families*			
Baseline self-completion questionnaire	x	x	x
Post-intervention self-completion questionnaire	x	x	x
*Data collected from children’s centres*			
Baseline manager/staff questionnaire	x	x	x
Post-intervention manager/staff questionnaire	x	x	x
Facilitation contacts questionnaire at 1, 3, 4/5 and 8 months	x	x	
Facilitation contacts interview at 1, 3, 4/5 and 8 months	x	x	
Activity logs	x	x	x
Post-intervention facilitation questionnaire	x	x	
Post-intervention facilitation interview	x	x	
Post-intervention implementation fidelity questionnaire	x	x	
Post-intervention implementation fidelity interview	x	x	

*Note*: post intervention is 12 months post commencement of the intervention in the IPB plus facilitation and IPB only arms and at 12 months post randomisation in the control arm.

### Ascertaining family outcomes

The baseline self-completion questionnaire will include questions on socio-demographic and economic characteristics, information about the household, previous fire-related injuries, fire safety behaviours and fire safety equipment, knowledge and understanding of what causes fires, home safety information provided by children’s centres and satisfaction with this information. Questions on fire safety behaviours and fire safety equipment will be based on those used in structured interviews of parents attending children’s centres in the four trial sites previously undertaken to inform the trial [24]. This questionnaire will be piloted on families attending children’s centres not taking part in the trial.

The post-intervention questionnaire will adapt the baseline questionnaire to include questions about resource use and expenditure incurred as a result of the intervention or relevant sessions offered by the control centres; i.e., travel costs to attend educational sessions, equipment purchased and services attended (e.g. smoking cessation). Shorter versions of the questionnaire will be used for reminders; up to two reminders will be used. A range of methods will be used for questionnaire administration including postal, face-to-face completion with children’s centre staff or researchers or telephone completion. Families completing questionnaires will be provided with a £5 gift voucher for use in local stores as small monetary incentives have been shown to increase response rates in a previous systematic review [26,27].

### Ascertaining children’s centre outcomes

Information will be collected from children’s centres managers or staff on the promotion of fire prevention using a postal questionnaire at baseline and post intervention in the IPB plus facilitation arm and the IPB only arm, and at baseline and 12 months post randomisation in the control arm. Questions will be based on those used in a national survey of injury prevention activity amongst children’s centres in England [28]. Questions concerning resource use related to implementing the IPB will be included in the post-intervention questionnaire for all arms of the trial. The baseline questionnaire was piloted on 10 children’s centres across England who were not selected to take part in the trial and minor modifications were made.

During the intervention period, data on process measures such as the number of fire prevention sessions provided by the children’s centre, number of centres with a written plan for implementing the IPB, number of staff trained in delivering the IPB per centre, number of specific fire safety sessions from the IPB provided for parents and number and type of other methods used by each centre to reach parents with fire safety messages (e.g. home visits by outreach workers, incorporation of fire safety messages in existing activities) will be ascertained from the IPB plus facilitation arm through the facilitation contacts questionnaire and interviews and the activity logs.

Post-intervention, information on the implementation of the IPB will be ascertained from an implementation fidelity questionnaire and from implementation fidelity interviews, with centre managers and/or staff responsible for the delivery of the IPB in the IPB plus facilitation and the IPB only arms of the trial. The questionnaire and interview schedule will be based on the Carroll framework [21], on the review of the literature on the implementation and facilitation of health promotion interventions undertaken for the trial, the systematic review of barriers and facilitators to home injury prevention [11] and findings from interviews of children’s centre managers and staff described above. Data collected from the range of outcome measurement tools will be mapped onto the PARIHS framework [20] (described above) to explore the success of the implementation process.

### Sample size

Eleven children’s centres per arm are required to detect an absolute difference in the percentage of families with a fire escape plan of 20% in either of the two intervention arms compared with the control arm. This assumes a control arm prevalence of 42% ascertained from a structured interview completed by families attending children’s centres in the four study centres [24], an intraclass correlation coefficient of 0.05, that outcomes are assessed on 20 families per children’s centre and is for 80% power and a 5% significance level (2-sided). This gives a total of 33 children’s centres. We will recruit a total of 36 children’s centres (9 per trial site), which will also allow for potential drop-out of one children’s centre per trial arm. Allowing for 33% loss to follow-up among families, 30 families per children’s centre will be recruited, a total of 1080 families.

### Randomisation

Once 30 families have been recruited at a children’s centre, centres will be stratified by trial site (4 strata: Nottingham, Bristol, Norwich, Newcastle) and randomly allocated within strata to one of three arms using permuted block randomisation, with a block size of 3. The allocation schedule will be produced by an independent statistician, using the randomisation algorithm in Stata. These lists will be provided to an independent administrator who will prepare sequentially numbered opaque envelopes (one set for each of the four trial coordinating centres) with these allocations.

Children’s centres will be randomised in trios, once each stratum contains 3 children’s centres. In this way, allocation will be concealed from the children’s centres and the researchers up to the point of actually randomising a trio of centres. Once a stratum contains 3 children’s centres, the administrator will open the envelopes for each block of 3 children’s centres, record the allocations and notify each of the trial coordinating centres of the allocation. Children’s centres will then be notified of their allocation by email.

### Blinding

It is not possible to blind children’s centre managers and staff or those providing the intervention to treatment arm allocation. As with many public health interventions, it is not possible to blind families to receipt of the intervention and as outcomes are self-reported, data on these cannot be collected blind to treatment arm allocation. If parents require support from a researcher to complete the follow-up questionnaire the researcher will not be blind to children’s centre treatment arm. Analyses will be undertaken blind to treatment arm allocation.

### Withdrawals

Participants will be free to withdraw from the trial at any stage but their data will be included up to the date of withdrawal. However, in order to minimise losses to follow-up, every effort will be made to maintain contact with families, even if they move to an area covered by a different children’s centre.

### Analysis

Baseline characteristics will be described for the three treatment arms using frequencies and percentages for categorical variables and with means and standard deviations for continuous variables if normally distributed or, if not, with medians and interquartile ranges.

All analyses of primary and secondary outcomes will be conducted on an intention-to-treat basis in that families and children’s centres will be analysed in the group to which they were randomised, regardless of whether they received or delivered the intervention. Analyses will be undertaken blind to treatment arm allocation. Statistical significance will be assessed based on likelihood ratio tests with a p value of <0.05 taken as significant. Analyses will be undertaken using Stata.

### Primary outcome measure

The proportion of families who report that they have a fire escape plan will be compared between treatment arms (IPB plus facilitation versus control and IPB only versus control) using random effects logistic regression to estimate odds ratios and 95% CI. The model will include randomisation stratum (trial site: Nottingham, Bristol, Norwich, Newcastle) as a fixed effect and will adjust for three cluster level variables (lead agency of the children’s centre (Local Authority, NHS or Voluntary sector led), Ofsted report scores for overall effectiveness and capacity for sustained improvement [29]) and two family level variables (having a fire escape plan at baseline and the Index of Multiple Deprivation (IMD) 2010 score [14]). These variables were selected as they are considered likely to be strongly associated with the outcome variable. The main comparisons will be between the IPB plus facilitation group versus control and the IPB only group versus control; a significance level of 0.05 will be used for each of these comparisons. Sub-group analyses will explore differential effects of the interventions by IMD, by adding interaction terms to the regression model. The intraclass correlation coefficient with a 95% CI will be estimated from the random effects regression model.

### Secondary outcome measures

#### Family level outcome measures

Binary secondary outcomes will be compared between treatment arms (IPB plus facilitation versus control and IPB only versus control) using random effects logistic regression and ordinal outcomes will be compared using random effects ordinal regression. Continuous outcomes will be compared using random effects linear regression, where assumptions are met. Where assumptions are not met, we will examine the distribution of the continuous outcome measure and categorise appropriately and analyse with ordinal or logistic models. Models will include randomisation stratum as a fixed effect and will also adjust for three cluster level variables (the lead agency of the children’s centre (Local Authority, NHS or Voluntary sector led [29]), and Ofsted report scores for overall effectiveness and capacity for sustained improvement) and two family level variables (baseline value of the secondary outcome measure, IMD).

### Children’s centre level outcome measures

The proportion of children’s centres providing information and advice on fire prevention (the 5 fire safety messages from the IPB, see the background section for details) will be compared between the IPB plus facilitation arm and the control arm and the IPB only arm and the control arm using logistic regression. These analyses will adjust for stratum, the lead agency of the children’s centre and Ofsted report scores for overall effectiveness and capacity for sustained improvement [29].

The proportion of children’s centres using methods other than the IPB to promote fire safety will be compared between the IPB plus facilitation arm and the IPB only arm using logistic regression. This analysis will adjust for stratum, the lead agency of the children’s centre and Ofsted report scores for overall effectiveness and capacity for sustained improvement.

The number of fire safety sessions provided will be compared between the IPB plus facilitation and the control arm and between the IPB only and control arm using linear regression if assumptions are met. Where assumptions are not met, we will examine the distribution of the number of fire safety sessions provided and categorise appropriately and analyse with ordinal or logistic models. These analyses will adjust for stratum, the lead agency of the children’s centre and Ofsted report scores for overall effectiveness and capacity for sustained improvement.

Barriers and facilitators to children’s centres implementing the IPB will be coded and categorised and described for the IPB plus facilitation and IPB only arms.

### Missing data

The main analysis will be a complete case analysis. If outcome measures have missing values at follow-up or at baseline the participants will be excluded from the analysis. A range of sensitivity analyses will be conducted to assess the robustness of the findings to missing data, these will include multiple imputation and analyses assuming no change in the primary outcome compared with the baseline value in families lost to follow-up [30].

### Health economic analysis

Cost-effectiveness will be determined by comparing cost differences between treatment arms with differences in the number of families in each group with a fire escape plan. This will be assessed by combining resource-use data collected throughout the trial with unit cost information and summed to obtain an average cost per person (together with its uncertainty) for each intervention. Secondly, these cost data will be combined with the clinical effectiveness data (eg., proportion of families who have a fire escape plan) to inform the incremental cost-effectiveness analysis. A sensitivity analysis will be undertaken to assess the robustness of the results to any assumptions made in the analysis.

### Qualitative analysis

Data from the telephone and face-to-face facilitation contacts between children’s centres and researchers will be analysed manually using content analysis after categorisation into main sub-headings [31]. A thematic analysis will then be conducted. Data from the 12 month implementation fidelity interviews will be managed using a qualitative data management package e.g. QSR NVIVO and analysed using Framework Analysis [32]. This is a structured method of qualitative data analysis where a priori themes are identified but emergent themes can also be identified and incorporated into the analytic framework. These themes will be agreed by researchers from the four trial sites and lay research advisors through an initial analysis of eight interviews, selected to represent a range of children’s centres. This framework will be used to undertake the analysis of the rest of the interviews, with further adaptation if required.

### Risks

We do not anticipate that participants will be exposed to any excess risk as a result of participating in this trial.

### Ethical and organisational review

The trial protocol was approved by the Derbyshire Research Ethics Committee (11/EM/0011) and the University of the West of England Bristol Research Ethics Committee (HSC/11/06/61). The trial received NHS organisational approval from Primary Care Trusts (PCTs) where staff who work in Children’s centres are employed by PCTs. Approval was provided by the following PCTs: Nottingham CityCare Partnership, Nottinghamshire Healthcare Trust (formerly NHS Nottinghamshire County), Bassetlaw PCT, NHS Bristol, North Somerset PCT, South Gloucestershire PCT, Norfolk PCT, Great Yarmouth & Waveney PCT, Northumbria Healthcare Foundation NHS Trust, Northumberland Healthcare Trust.

## Discussion

This trial will enable an evaluation of the effectiveness and cost-effectiveness of implementing an IPB in children’s centres for promoting fire prevention. It will also explore and assess the process of implementing the IPB using a range of methodologies, including a nested qualitative study. The intervention we have developed and are testing within this trial is a complex intervention [15] and one that children’s centres can tailor to suit their individual circumstances. Substantial preparatory work has been undertaken as part of the development of the intervention, including a review of implementing health promotion interventions to identify appropriate theoretical models, interviews with key informants and workshops with stakeholders including children’s centre managers and staff, the Fire and Rescue Service, NHS staff and commissioners of children’s services. In addition, a series of systematic reviews updating the evidence on what works in injury prevention and identifying barriers and facilitators to home injury prevention as well as surveys and interviews with children’s centre managers and staff and with parents of pre-school children to identify barriers and facilitators to injury prevention have been undertaken and their findings incorporated into the design of the trial. Our evaluation includes multiple methods to quantify and explore implementation and a range of outcome measures covering family and children’s centres outcomes, including economic outcomes.

As there is a steep social gradient in fire-related thermal injuries we considered it important to test the effectiveness of our intervention in children’s centres situated in the more disadvantaged areas in each trial site. However, families attending children’s centres in these areas may have lower levels of literacy and families with young children are likely to be more mobile than other families [33]. This may pose challenges for trial recruitment and retention, so we are using a range of recruitment and follow-up methods including small monetary incentives.

This trial will be the first randomised controlled trial to provide data on the effectiveness of implementing an IPB in children’s centres to promote fire prevention in disadvantaged populations. Findings should enhance the evidence base for fire prevention for young children. This information will be relevant for policy makers, commissioners of injury prevention services, Fire and Rescue Services, children’s centre staff and other injury prevention practitioners.

## Competing interests

The author(s) declare that they have no competing interests.

## Authors’ contributions

DK had the original idea for the wider programme of work, this particular trial, obtained NIHR funding and helped with drafting the protocol. TD led on drafting the protocol and ET, EM, RR, AS, CC, NC, JS, MH, EP and MW helped with drafting the protocol. All authors read and approved the final manuscript.

## Pre-publication history

The pre-publication history for this paper can be accessed here:

http://www.biomedcentral.com/1471-2458/14/69/prepub

## References

[B1] Peden M Oyegbite K Ozanne-Smith J World Report on Child Injury Prevention 2008 Geneva: World Health Organisation 26269872

[B2] Sethi D Towner E Vincenten J Segui-Gomez M Racioppi F European Report on Child Injury Prevention 2008 Geneva: World Health Organisation

[B3] Department for Communities and Local Government Fire statistics Great Britain 2011 to 2012 2012 London: Department for Communities and Local Government

[B4] Mulvaney C Kendrick D Towner E Brussoni M Hayes M Powell J Robertson S Ward H Fatal and non-fatal injuries in England 1995–2004: time trends and inequalities by age, sex and area deprivation J Public Health 2008 31 1 154 161 10.1093/pubmed/fdn103 19074453

[B5] Edwards P Roberts I Green J Deaths from injury in children and employment status in family: analysis of trends in class specific death rates Br Med J 2006 333 119 121 10.1136/bmj.38875.757488.4F 16829537 PMC1502180

[B6] Runyan CW Bangdiwala SI Linzer MA Sacks JJ Butts J Risk factors for fatal residential fires N Engl J Med 1992 327 12 859 863 10.1056/NEJM199209173271207 1508246

[B7] Marshall SW Runyan CW Bangdiwala SI Linzer MA Sacks JJ Butts JD Fatal residential fires: who dies and who survives? J Am Med Assoc 1998 279 20 1633 1637 10.1001/jama.279.20.1633 9613913

[B8] Kendrick D Young B Mason-Jones AJ Ilyas N Achana FA Cooper NJ Hubbard SJ Sutton AJ Smith S Wynn P Mulvaney C Watson MC Coupland C Home safety education and provision of safety equipment for injury prevention Cochrane Database Syst Rev 2012 Issue 9 Art. No.: CD005014. DOI:10.1002/14651858.CD005014.pub3 10.1002/14651858.CD005014.pub3 PMC9758703 22972081

[B9] DiGuiseppi C Goss CW Higgins JPT Interventions for promoting smoke alarm ownership and function Cochrane Database Syst Rev 2001 Issue 2 Art. No.: CD002246. DOI:10.1002/14651858.CD002246 10.1002/14651858.CD002246 PMC7017850 11406039

[B10] The Audit Commission, Health Care Commission Better Safe than Sorry: Preventing Unintentional Injury to Children 2007 London: The Audit Commission

[B11] Ingram JC Deave T Towner E Errington G Kay B Identifying facilitators and barriers for home injury prevention interventions for pre-school children: a systematic review of the quantitative literature Health Educ Res 2011 27 2 258 268 doi:10.1093/her/cyr066 21873613 10.1093/her/cyr066 PMC3529629

[B12] Department for Education http://www.foundationyears.org.uk/wp-content/uploads/2011/10/Core_purpose_of_Sure_Start_Childrens_Centres.pdf 2012 London: Department for Education http://media.education.gov.uk/assets/files/pdf/s/sure%20start%20childrens%20centres%20core%20purpose.pdf

[B13] Department for Education Sure Start Children’s Centres Statutory Guidance 2010 London: Department for Education

[B14] The English Indices of Deprivation 2010 (IMD 2010) 2012 http://www.communities.gov.uk/publications/corporate/statistics/indices2010

[B15] Craig P Dieppe P Macintyre S Michie S Nazareth I Petticrew M Developing and evaluating complex interventions: the new Medical Research Council guidance Br Med J 2008 337 979 983 ISSN 0959–8138 10.1136/bmj.a979 PMC2769032 18824488

[B16] Cooper NJ Kendrick D Achana F Dhiman P He Z Wynn P le Cozannet E Saramago P Sutton AJ Network Meta-analysis to Evaluate the Effectiveness of Interventions to Increase the Uptake of Smoke Alarms Epidemiol Rev 2012 34 1 32 45 DOI:10.1093/epirev/mxr015 10.1093/epirev/mxr015 22128085

[B17] Saramago P Methodological Issues in the Analysis of Individual- and Aggregate-Participant Level Data for Cost Effectiveness Analysis PhD thesis 2012 York: University of York

[B18] Kelly M Swann C Evidence into practice and health inequalities Health Educ 2004 104 5 269 271 10.1108/09654280410560514

[B19] Brussoni M Towner E Hayes M Evidence into practice: Combining the art and science of injury prevention Inj Prev 2006 12 6 373 377 PMID: 17170184 10.1136/ip.2005.011403 17170184 PMC2564413

[B20] Rycroft-Malone J The PARIHS framework – a framework for guiding the implementation of evidence-based practice J Nurs Care Qual 2004 19 297 304 10.1097/00001786-200410000-00002 15535533

[B21] Carroll C Patterson M Wood S Booth A Rick J Balain S A conceptual framework for implementation fidelity Implement Sci 2007 2 40 DOI: 10.1186/1748-5908-2-40 10.1186/1748-5908-2-40 18053122 PMC2213686

[B22] Gielen AC Sleet D Application of behavior-change theories and methods to injury prevention Epidemiol Rev 2003 25 65 76 10.1093/epirev/mxg004 12923991

[B23] Watson M Kendrick D Coupland C Validation of a home safety questionnaire used in a randomised controlled trial Inj Prev 2003 9 180 183 10.1136/ip.9.2.180 12810749 PMC1730946

[B24] Deave T Goodenough T Stewart J Towner E Majsak-Newman G Hawkins A Coupland C Kendrick D Contemporary hazards in the home: keeping children safe from thermal injuries Arch Dis Child 2014 98 7 485 489 doi:10.1136/archdischild-2012-302901 23592729 10.1136/archdischild-2012-302901

[B25] NICE Strategies to prevent unintentional injuries among under-15s: guidance 2010 NICE Public Health Guidance, PH 29 http://guidance.nice.org.uk/PH29/Guidance/pdf/English

[B26] Edwards PJ Roberts I Clarke MJ DiGuiseppi C Wentz R Kwan I Cooper R Felix LM Pratap S Methods to increase response to postal and electronic questionnaires Cochrane Database Syst Rev 2009 Issue 3 Art. No.: MR000008. DOI:10.1002/14651858.MR000008.pub4. Editorial Group: Methodology Review Group. Last assessed as up-to-date: December 10. 2008 10.1002/14651858.MR000008.pub4 PMC8941848 19588449

[B27] McColl E Jacoby A Thomas L Soutter J Bamford C Steen N Design and use of questionnaires: a review of best practice applicable to surveys of health service staff and patients Health Technol Assess 2001 5 31 10.3310/hta5310 11809125

[B28] Watson M Mulvaney C Kendrick D Stewart J Coupland C Hayes M Wynn P National survey of the injury prevention activities of children’s centres Health Soc Care Community 2014 22 1 40 46 Doi:10.1111/hsc.12059 10.1111/hsc.12059 23837887 PMC3920631

[B29] Ofstedhttp://www.ofsted.gov.uk/inspection-reports/find-inspection-report

[B30] White I Strategy for intention to treat analysis in randomised trials with missing outcome data Br Med J 2011 342 d40 10.1136/bmj.d40 21300711 PMC3230114

[B31] Mays N Pope C Ziebland S Pope C, Mays N Analysing Qualitative Data Qualitative Research in Health Care 2000 London: BMJ Books 75 88 10.1136/bmj.320.7227.114 PMC1117368 10625273

[B32] Ritchie J Spencer L Bryman A, Burgess R Qualitative Data Analysis for Applied Policy Research Analyzing Qualitative Data 1994 Oxford: Routledge

[B33] Institute of Health Equity An Equal Start. Improving Outcomes in Children’s Centres: an Evidence Review 2012 London: University College London

